# Protocol for contactless electric current sensing, processing, and storage using a drone-integrable sensor

**DOI:** 10.1016/j.xpro.2025.104096

**Published:** 2025-09-17

**Authors:** Khaled Osmani, Detlef Schulz

**Affiliations:** 1 Department of Electrical Engineering, Helmut Schmidt University, 22043 Hamburg, Germany

**Keywords:** Energy, Physics

## Abstract

Here, we present a protocol for implementing a contactless, drone-mounted current sensor to measure and record electric current norms in overhead transmission lines. We describe steps for designing, fabricating, and assembling the printed circuit board (PCB) and programming the Arduino Mega 2560 as the data processor. We further outline the integration of MATLAB scripts for graphical visualization of the sensed currents, ensuring efficient data interpretation. This protocol enables accurate laboratory-based measurements up to 98.3% under high-distortion test scenarios.

For complete details on the use and execution of this protocol, please refer to Osmani et al.^1^

## Highlights

• Procedures for implementing the corresponding analog signal processing units
• Instructions for programming the Arduino Mega 2560 as a data processor
• Guidance on visualizing results in MATLAB with date and timestamps

## Abstract (editor highlights)

Publisher’s note: Undertaking any experimental protocol requires adherence to local institutional guidelines for laboratory safety and ethics.

## Graphical abstract

**Figure:**
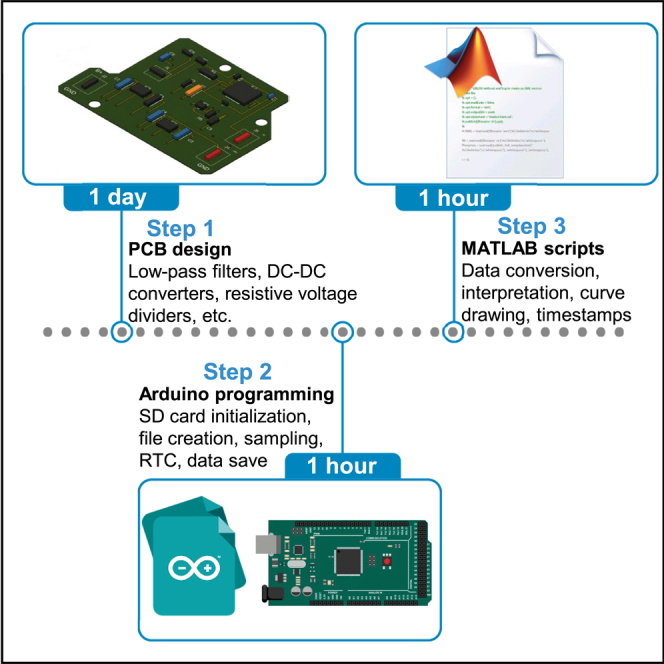

## Before you begin

This protocol outlines a comprehensive approach for developing a drone-mounted electrical sensor designed for contactless current measurement in overhead Transmission Lines (TLs). The sensor system consists of an encapsulating unit (i.e., sensor box) that houses dedicated Signal Processing Units (SPUs). These SPUs process the captured Magnetic Fields (MFs) generated by the current flowing through the TL and convert them into computer-readable data. The resulting voltage signals are then fed into an Arduino Mega 2560. Using a Real-Time Clock (RTC)-based circuit, these sensed voltages accurately reflect the original current-inducing signals and are subsequently stored on a micro–Secure Digital (SD) card along with corresponding timestamps. The primary objective is to facilitate the visualization of the Root Mean Square (RMS) values of the recorded current at any given time. To achieve this, the protocol further demonstrates how to generate MATLAB plots of the obtained results once the microSD card is inserted into a computer with MATLAB installed.

### Scope and structure

This protocol addresses the design, implementation, and evaluation of a drone-deployable sensor system for non-invasive, time-resolved current measurements in overhead TLs. The sensor functions as a drone payload and is designed to mechanically attach itself to the target TL during deployment. A specialized housing material allows uninterrupted penetration of MFs while withstanding the mechanical impact associated with repeated deployment events. Internally, the sensor unit integrates Hall-effect magnetic field sensors (i.e., a sum of 8 MF sensors, with 4 installed in each of the adjacent tube of the sensor box), SPUs, and a microcontroller-based acquisition system capable of continuous operation for up to six hours per charge. Captured data is automatically timestamped and stored locally for subsequent analysis. The manuscript is organized into sequential sections detailing: a) the analog circuitry, including filtering and power regulation; (b) the microcontroller programming for data acquisition and storage; and c) post-deployment data processing and visualization using MATLAB.

The protocol systematically details each step of the process, beginning with the design of the analog circuitry, including Low-Pass Filters (LPFs), Direct Current (DC)-DC converters, and voltage dividers. It then presents the programming methodologies required to convert the measured MFs into corresponding voltages and reliably store them on the microSD card. Finally, the protocol provides a MATLAB script for graphical representations of the recorded current values (from the processed voltage signals). Measuring current in overhead transmission lines (TLs) is inherently complex and has traditionally required dedicated control stations along with substantial expertise,^2^ particularly following the widespread integration of renewable energy resources.^3^ Although recent developments in energy management systems^4^ and Internet of Things (IoT)-based microgrid monitoring techniques^5^ offer promising directions, much of the existing research in this field continues to rely on less dynamic and inflexible methodologies for offline current monitoring.^6^^,^^7^^,^^8^ In contrast, the proposed protocol introduces an innovative and non-invasive approach to current measurement using a drone-deployed sensor prototype. The system is designed to function as a payload on an autonomous drone, which releases the sensor onto the TL. Once the sensor is retrieved after deployment, a history of recorded current values can be graphically visualized. As a result, this protocol offers a practical solution for current measurement applications, particularly in remote monitoring and smart grid systems.

**Figure 1:**
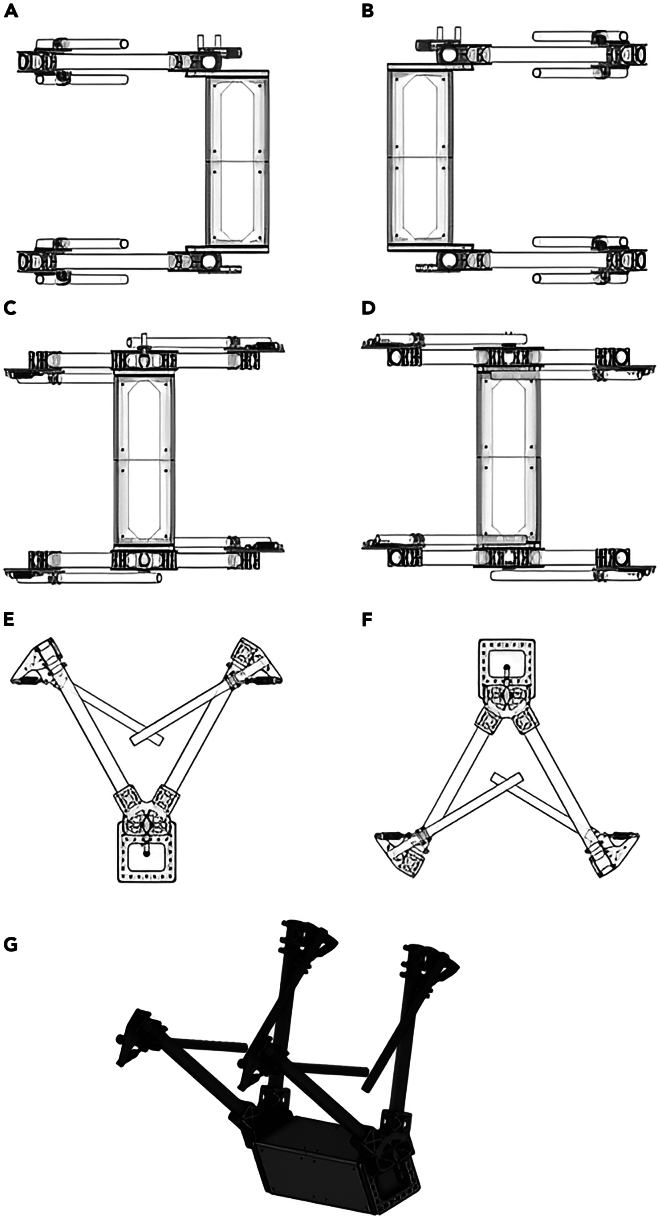
Computer-aided design of the sensor box Computer aided design of the sensor box with side views from (A) front, (B) back, (C) bottom, (D) top, (E) right, (F) left, and (G) 3D right tilted 45°.

**CRITICAL:** The protocol is applicable to the sensor box depicted in Figure 1, which possesses the specified material characteristics.

***Note:*** Since the operation of the sensor proposed in this protocol relies entirely on MF capture, it is recommended to use a sensor box similar to the one shown in Figure 1. This ensures compliance with the sensor's operational workflow (i.e., repeated deployment and retrieval via drone on the TLs) while maximizing MF penetration for optimal signal processing.

1. Use a Carbon Fiber (CF) based material to fabricate the sensor box design of Figure 1 with a low magnetic permeability ≪ 1 H/m, high compressive strength ≥600 MPa, high tensile strength ≥ 3500 MPa, and high tensile modulus ≥230 GPa.

***Note:*** The geometric structural architecture should avoid all forms of bending, flexing, and curving.

***Note:*** The base structure (i.e., prism) with dimensions of 90 mm × 300 mm × 90 mm is designed to encapsulate all SPUs, along with the Arduino Mega 2560, the power supply battery, and the necessary cables and connectors.

2. Maintain a stable distance between the embedded MF sensors and the overhead TL by securing the fastening tubes (with radii of 8 mm and 12.5 mm) in place.
3. Install each MF sensor inside the two adjacent (4 MF sensors in each) fastening tubes with radii of 12.5 mm using detachable cylindric barrels.
4. Install caps on the tops of the other tubes (i.e., those with radii of 8 mm and the remaining two tubes with radii of 12.5 mm) to prevent the penetration of water and debris.

***Note:*** The sensor box overall weight should not exceed 0.6 Kg.

**Figure 2:**
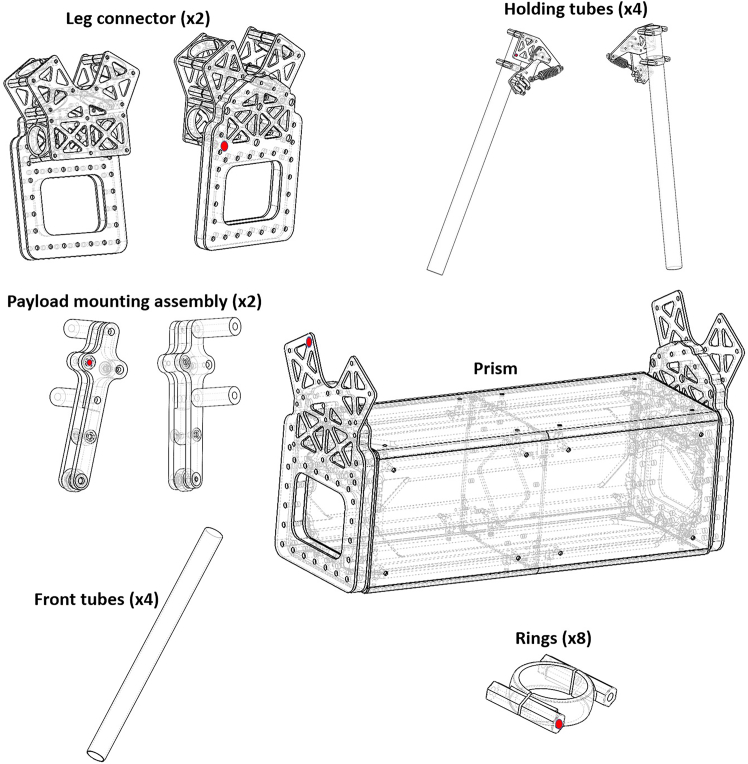
The six mechanical components comprising the sensor box assembly

***Note:*** The sensor box shown in Figure 1 represents a mechanical assembly composed of six distinct geometrical components, joined using 4 mm screws and corresponding lock nuts. Each mechanical part illustrated in Figure 2 is individually connected to the others via screws and mechanical joints. The central prism depicted in Figure 2 serves as the core housing for all electronic components. Leg connectors are affixed to the prism through pre-drilled holes (some of which are highlighted in red in the various shapes of Figure 2) using 4 mm screws. Once the leg connectors are secured to the prism, the holding tubes on both the left and right sides can be mounted and fastened using the same type of screws. Two rings are inserted onto each front tube, enabling their connection to the holding tubes through aligned holes, also secured with 4 mm screws. Overall, the sensor enclosure constitutes a mechanically integrated assembly of the various components shown in Figure 2, all of which were designed in SolidWorks.

**Figure 3:**
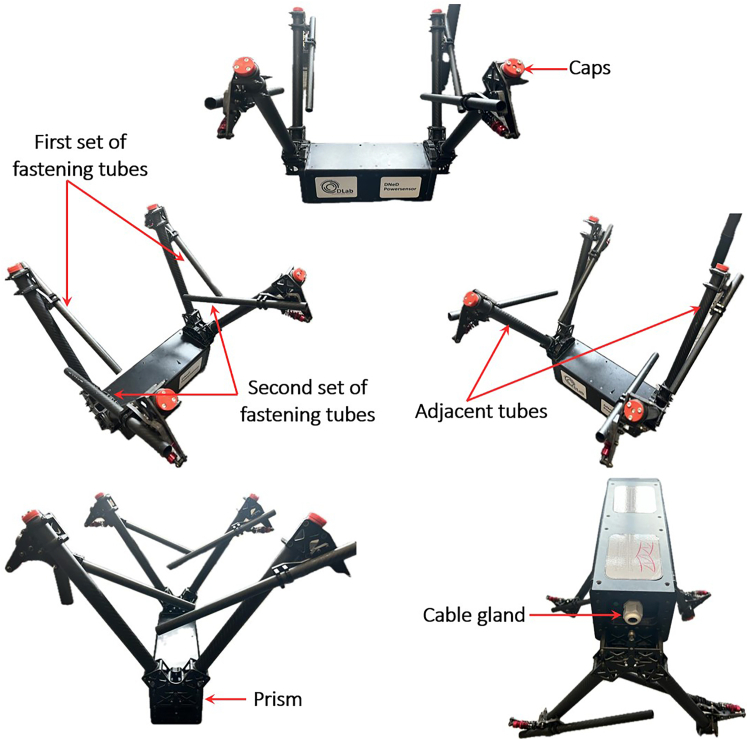
Physical overview of the proposed sensor box and its constituent components

***Note:*** The conceptual design of the sensor box, illustrated in Figure 1, has been physically constructed as shown in Figure 3. The enclosure integrates all the circuitry required for the implementation of contactless current sensing. This includes the MF sensors, the battery, SPUs, and all associated electronic components.

***Note:*** The caps shown in Figure 3 are designed to prevent the ingress of water and debris when the sensor box is suspended above the TL under rainy conditions. The sensor box accommodates two possible orientations for attachment to the TL upon deployment from the drone. To support this dual-orientation design, two sets of fastening tubes are integrated, ensuring secure fixation regardless of the side to which the box adheres. The MF sensors are mounted in adjacent tubes, corresponding to the two potential fixation orientations. When the sensor box is secured via the first set of fastening tubes, the MF sensor located in the nearby tube becomes active for current measurement. The same applies to the second set of fastening tubes, ensuring reliable sensing in both configurations. Within the prism section shown in Figure 3, all SPUs, along with the Arduino controller and battery, are housed. This compartment offers the largest available space in the sensor box, encapsulating all required circuit components and their associated wiring. The output cables from each MF sensor are routed into the prism through the cable gland illustrated in Figure 3.

### Innovation

The presented protocol advances beyond existing grid’s monitoring methods^4^^,^^5^^,^^6^^,^^7^^,^^8^ by introducing an innovative, drone-deployable sensor box specifically designed for offline current monitoring in overhead TLs. The device is constructed to operate robustly under environmental and mechanical challenges while functioning as a self-contained data repository. Its architecture enables direct storage of current measurements up to 6 kA, in second-wise measurements over a 6-h duration, which can subsequently be used to generate accurate current–time curves in MATLAB without additional intermediate processing. This unique combination of portability, resilience, and measurement fidelity represents a substantive departure from existing techniques. By enabling precise current monitoring in challenging field conditions without reliance on continuous communication or fixed infrastructure, the proposed protocol establishes a practical and scalable alternative for power system applications.

## Key resources table

**Table:** 

REAGENT or RESOURCE	SOURCE	IDENTIFIER
**Software and algorithms**		

MATLAB R2023a	MathWorks	https://de.mathworks.com/products/matlab.html
Autodesk EAGLE	Autodesk	https://www.autodesk.com/products/eagle/overview
SolidWorks 2022	Dassault Systèmes SolidWorks Corporation	https://www.solidworks.com/
Arduino IDE	Arduino.cc	https://www.arduino.cc/en/software

**Other**		

Tubes’ caps	Helmut Schmidt University central workshop	N/A
Internal barrels	Helmut Schmidt University central workshop	N/A
Cable shielding	Helmut Schmidt University central workshop	N/A
Entire CF sensor box housing (including the prism, fastening tubes, and connectors)	Emqopter GmbH	N/A
Screws of different widths (4, 5, and 6 mm)	Helmut Schmidt University central workshop	N/A
Custom-designed PCB	This paper	N/A
Shielded twisted pair cable	M5Stack Technology Co., Ltd.	A088-B
Magnetic field sensor	Texas Instruments	DRV5055A1
SMD multilayer ceramic capacitor	KEMET	C1206X104J8HACTU
Wire-to-board terminal block	Molex	39773-003
Wire-to-board terminal block	PHOENIX CONTACT	1729128
Active filter	ANALOG DEVICES	MAX280CWE+
Silicon oscillator	ANALOG DEVICES	LTC6900CS5#TRMPBF
LDO voltage regulator (5V)	ROHM Semiconductor	BD50GA5MEFJ-LBH2
LDO voltage regulator (8V)	ROHM Semiconductor	BD80GC0VEFJ-ME2
Tantalum capacitor	KYOCERA AVX	TAJW225K050RNJ
SMD chip resistor	Panasonic	ERA-8AEB364V
SMD chip resistor	Panasonic	ERA-8ARW2942V
Multilayer ceramic capacitor	Vishay	VJ0805Y154JXXTW1BC
Tantalum capacitor	KYOCERA AVX	TAJA106J010RNJ
SMD metal foil resistor	Vishay Precision Group	Y112125K0000T9R
SMD thin film resistor	KOA Speer	RN73H1JTTD3523D100
3S1P (12 V) 5,000 mAh battery	ENERPROF	https://enerprof.de/products/enerpower-3s1p-11-1v-akku-12v-5000mah-55wh-open-end-kabel-3x1
Arduino Mega 2560	Arduino.cc	A000067
SPI microSD card module	kwmobile	N/A
microSD card	SanDisk	WDD032G1P0C
Peripheral module	ANALOG DEVICES	DS3231MPMB1#
Pluggable terminal block	WAGO	221-2411
Pushbutton switch illuminated	EAO	82-6851.1134

## Step-by-step method details

### PCB design, fabrication, and full assembly

**Timing: 1 day**

For this major step, the Printed Circuit Boards (PCBs) are designed, acting as signal transducers between the output signals of the DRV5055A1 sensors and the Arduino Mega 2560.

**Figure 4:**
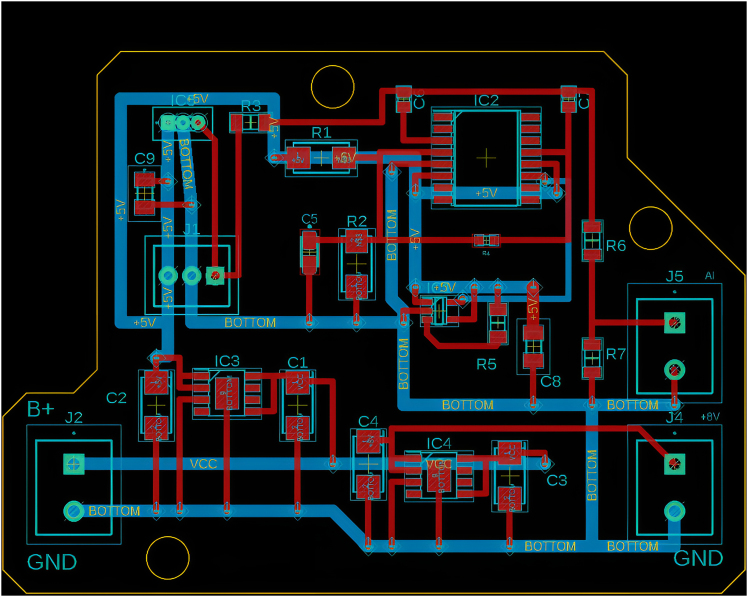
Board design overview of the PCB

**Figure 5:**
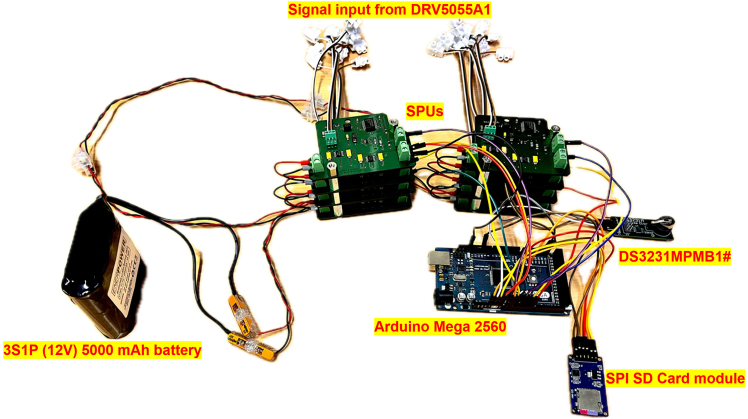
Overview of the entire circuit used for current sensing and data saving

1. Using Autodesk EAGLE.
  a. Visit the Autodesk EAGLE free-download website http://www.autodesk.com/products/eagle/free-download.
  b. Select the installer appropriate for your operating system (e.g., Windows).
  c. Verify that your computer meets the minimum system requirements listed on the website.
  d. Run the download installer and follow the on-screen instructions.
  e. Accept the license agreement when prompted.
  f. Complete the installation process.
  g. Launch Autodesk EAGLE.
  h. Sign in with an existing Autodesk account, or create a free account if you do not already have one.
    ***Note:*** Once signed in, the software will activate the free personal-use license (alternatively you can apply any professional license associated with your account).
  i. Start a new project (e.g., Schematic).
  j. Navigate to the Control Panel.
  k. Right-click on the Projects folder and select New Project to create a new project workspace.
    ***Note:*** This free version of EAGLE, available through Fusion 360 for personal use, imposes limitations of 2 schematic sheets, 2 signal layers, and a board area of 80 cm^2^. Despite these restrictions, it is still possible to create the PCBs for this protocol using the free version, which does not require a subscription for commercial use. Users can import libraries, design custom boards, and generate all necessary data for PCB fabrication (e.g., Gerber files, drill files, and board outlines).
2. Creating and integrating electronic components in Autodesk EAGLE.
  ***Note:*** Please refer to the troubleshooting 1 and 2 to acknowledge the probabilistic failures associated in this step with their potential solutions.
  a. Designing DRV5055A1 in Autodesk EAGLE.
    i. Open EAGLE 9.6.2 control panel.
    ii. Open Library Editor and create a new Symbol (e.g., DRV5055A1).
    iii. Draw rectangle, add corresponding label pins (VCC, OUT, GND), then save.
    iv. Create a new footprint Package (TO-92), and place the 3 pads as per datasheet.
    v. Draw silkscreen outline, assign pad numbers then save.
    vi. Integrate the device by attaching its symbol and package.
    vii. Map pins to corresponding pads then save.
  b. Designing MAX280CWE+ in Autodesk EAGLE.
    i. Open Library Editor and create a new symbol.
    ii. Draw rectangle, add and label pins as per datasheet, then save.
    iii. Create a new footprint package (SOIC-16 Wide), and place 16 pads as per datasheet.
    iv. Draw silkscreen outline, assign pad numbers then save.
    v. Integrate the device by attaching its symbol and package.
    vi. Map pins to corresponding pads then save.
  c. Designing LTC6900CS5#TRMPBF in Autodesk EAGLE.
    i. Open Library Editor and create a new symbol.
    ii. Draw rectangle, add and label pins as per datasheet, then save.
    iii. Create a new footprint package (SOT-23-5), and place 5 pads as per datasheet.
    iv. Draw silkscreen outline, assign pad numbers then save.
    v. Integrate the device by attaching its symbol and package.
    vi. Map pins to corresponding pads then save.
  d. Designing BD50GA5MEFJ-LBH2 in Autodesk EAGLE.
    i. Open Library Editor and create a new symbol.
    ii. Draw rectangle, add and label pins as per datasheet, then save.
    iii. Create a new footprint package (HTSOP-J8), and place 9 pads as per datasheet.
    iv. Draw silkscreen outline, assign pad numbers then save.
    v. Integrate the device by attaching its symbol and package.
    vi. Map pins to corresponding pads then save.
  e. Designing BD80GC0VEFJ-ME2 in Autodesk EAGLE.
    i. Open Library Editor and create a new symbol.
    ii. Draw rectangle, add and label pins as per datasheet, then save.
    iii. Create a new footprint package (HTSOP-J8), and place 9 pads as per datasheet.
    iv. Draw silkscreen outline, assign pad numbers then save.
    v. Integrate the device by attaching its symbol and package.
    vi. Map pins to corresponding pads then save.
  f. Creating a Two-Terminal Device in Autodesk EAGLE (e.g., resistor, capacitor, connector, etc.).
    i. Open Library Editor, create a generic two-pin symbol, finally label terminals as “1” and “2”.
    ii. Create a new package in which you place two pads according to standard datasheet spacing and dimensions.
    iii. Draw a silkscreen outline to represent the component’s physical shape.
    iv. Integrate the symbol and package into a new device.
    v. Ensure correct pin mapping.
    vi. Save the library component.
      ***Note:*** Upon replicating this protocol, users must bear in mind the challenges behind the design of the referred components in Autodesk EAGLE, as such a process is time-consuming and prone to errors, especially in symbol-pin mapping, footprint accuracy, and ensuring overall compatibility with the PCB layout.
3. Circuit arrangement as EAGLE board
  ***Note:*** The wire connectors should be placed at the boarders (i.e., extremities) of each PCB to facilitate cable routing. The DC-DC converters should be placed at the bottom of each PCB (i.e., dedicating a common ground area). The LPF circuitry is to be centered on each PCB layout. The traces of control signals should be at minimum of 25 mils, and those for power of 50 mils.
  **CRITICAL:** Make sure that the design is designed in a two-layer configuration (i.e., top and bottom).
  a. Connect the output of the DRV5055A1 to ERA-8ARW2942V.
  b. Connect the other terminal of ERA-8ARW2942V to the FB input of MAX280CWE+ through the capacitor VJ0805Y154JXXTW1BC and to the OUT pin of the MAX280CWE+ through the other capacitor VJ0805Y154JXXTW1BC.
  c. Connect both terminals of VJ0805Y154JXXTW1BC to 1729128.
  d. Connect C1206X104J8HACTU between the VCC and GND of the DRV5055A1.
  e. Connect the GND of DRV5055A1 to TAJA106J010RNJ and Y112125K0000T9R and V- pin of the MAX280CWE+. Connect the other terminal of TAJA106J010RN and Y112125K0000T9R to AGND pin of the MAX280CWE+, Y112125K0000t9R and OUT pin of the MAX280CWE+ through RN73H1JTTD3523D100.
  f. Connect +5V to DIVIDER_RATIO of the MAX280CWE+ and to the floating terminal of Y112125K0000T9R.
  g. Connect Cosc pin of MAX280CWE+ to OUT pin of LTC6900CS5#TRMPBF.
  h. Connect V+ pin of MAX280CWE+ to DIV pin of LTC6900CS5#TRMPBF and C1206X104J8HACTU and ERA-8AEB364V. Connect the other terminals of C1206X104J8HACTU and ERA-8AEB364V to GND and SET pin of LTC6900CS5#TRMPBF respectively.
  i. Connect GND pin of LTC6900CS5#TRMPBF and GND pins of BD50GA5MEFJ-BH2 and BD80GC0VEFJ-ME2.
    **CRITICAL:** Make sure that all GND in the PCB are common, and connected to the GND of the 3S1P (12 V) 5000 mAh battery.
  j. Connect VCC and GND of 3S1P (12 V) 5000 mAh battery to Terminal 1 and Terminal 2 respectively of the 1729128 terminal blocks.
  k. Connect Terminal 1 of 1729128 to VCC pins of both BD50GA5MEFJ-LBH2 and BD80GC0VEFJ-ME2.
  l. Connect two TAJW225K050RNJ, one to VCC pin, other to Vo pin of both BD50GA5MEFJ-LBH2 and BD80GC0VEFJ-ME2.
  m. Connect the negative terminals of the four TAJW225K050RNJ and GND of BD50GA5MEFJ-LBH2 and GND of BD80GC0VEFJ-ME2 to the common GND of the entire circuit.
  n. Insert round circles on the board design dedicated for the bolts of 6 mm insertion.
    ***Note:*** The bolts act for enabling the vertical overlapping of each PCB (exists a total of 8 PCBs, each specific for each of the 8 MF sensors). The aim behind is to compress the size needed for the realization of the sensor, with the constrained spatial geometry architecture shown in Figure 1.
  o. Conduct Electrical Rule Check (ERC) and Design Rule Check (DRC) checks and make sure that the board is error free.
  p. Generate the required Computer-Aided Manufacturing (CAM) data for PCB manufacturing such as assembly, drill files, and “gerber” files. The complete circuit design should look like as depicted in Figure 4.
    ***Note:***Figure 4 shows a PCB layout, intended as a reference for creating compact and manufacturable boards within the limitations of the free version (specifically, two signal layers, two schematic sheets, and an 80 cm^2^ board area). The layout demonstrates efficient use of space by minimizing blank areas between SMD components while maintaining safe distances for reliable soldering. It provides an example of how components should be arranged and routed to achieve a dense yet functional design.
4. PCB fabrication.
  ***Note:*** Please refer to the troubleshooting 3, 4, and 5 to acknowledge the probabilistic failures associated in this step with their potential solutions.
  a. Import the “gerber”, drill, and netlist files into CAM350.
  b. Perform a full Design for Manufacturability (DFM) check using Valor New Product Introduction (NPI).
  c. Confirm that the track widths are of 50 mil for power connections, 25 mil for control.
  d. Confirm the existence of clearances.
  e. Confirm that the drill sizes are more than or equal to 0.3mm, and outline precision.
  f. Use an Orbotech Diamond 10 laser photoplotter to create high-resolution films for each copper layer, solder mask, and silkscreen, maintaining alignment accuracy within ±25μm.
  g. Clean FR-4 panels and apply photoresist using a roll laminator, then align photoplots and expose them with Ultra Violet (UV) light in an LPKF ProtoLaser LDI system.
  h. Develop and etch to form precise conductor patterns.
  i. Perform an Automated Optical Inspection (AOI) with an Orbotech Discovery Ⅱ 9000 AOI system.
  j. Stack inner layers with prepregs and cores and laminate them under controlled temperature and pressure using a Burkle multi-layer press.
  k. Drill all plated through-holes, vias, and mechanical holes using a Schmoll CNC drilling machine.
  l. Deposit electroless copper.
  m. Reinforce with electroplating in an Atotech metallization line, achieving approximatively 25 μm plating thickness.
  n. Repeat photoimaging on outer layers with the LPKF ProtoLaser LDI, electroplate copper traces using Atotech plating systems.
  o. Etch away unprotected copper in automatic etching lines, ensuring final copper thickness between 35–70 μm.
  p. Coat solder mask using a curtain coater.
  q. Expose and develop pad openings with a UV system.
  r. Print silkscreen legends with an Orbotech inkjet printer, keeping legend line widths at or above 0.15 mm.
  s. Apply the final surface finish via an Atotech ENIG line.
  t. Perform electrical continuity testing using an ATG Luther & Maelzer flying probe tester.
  u. CNC-profile the panels with an LPKF ProtoMat S104.
  v. Finalize with visual inspection and thickness measurement.
5. Soldering.
  ***Note:*** Surface-Mount Device Package (SMD) packages (e.g., MAX280CWE+) are better be soldered using a soldering machine. That is to say, manual soldering is to be avoided in order to avoid Integrated Circuit (IC) damaging, especially since the ICs used in this protocol are of small dimensions.
  ***Note:*** Please refer to the troubleshooting 6, 7, 8, and 9 to acknowledge the probabilistic failures associated in this step with their potential solutions.
  a. Solder paste application.
    i. Secure the PCB in the EKRA X5 stencil printer and ensure proper alignment.
    ii. Position a stainless-steel stencil over the PCB to match the IC pad locations.
    iii. Use a squeegee system to evenly spread solder paste across the stencil openings.
    iv. Lift the stencil carefully to leave precise solder paste deposits on the pads.
  b. Component placement.
    i. Load the PCB into the Mycronic MY300 pick-and-place machine.
    ii. Ensure correct component positioning via optical recognition and fiducial alignment.
  c. Reflow soldering by transferring the populated PCB onto the conveyor belt of the Heller 1809 MK5 reflow oven.
  d. Quality inspection.
    i. Use the CyberOptics SQ3000 AOI system to detect placement or soldering errors.
    ii. Perform X-ray inspection with the Nikon XT V 160 for Ball Grid Array (BGA) ICs to verify hidden joints.
    iii. Conduct electrical testing to confirm correct circuit functionality.
6. Full electronic assembly.
  a. Connect all GND of each DRV5055A1 to the GND of the Arduino Mega 2560 and to the common GND of the entire circuitry.
  b. Connect the GND of DS3231MPMB1# to the common GND of the circuit.
  c. Connect the Serial Clock Line (SCL, i.e., I^2^C serial clock pin) of the DS3231MPMB1# to the SCL pin of the Arduino Mega 2560 (i.e., pin number 21).
  d. Connect the Serial Data Line (SDA, i.e., I^2^C serial data pin) of the DS3231MPMB1# to the SDA pin of the Arduino Mega 2560 (i.e., pin number 20).
  e. Connect the GND of the Serial Peripheral Interface (SPI) microSD card module to the common GND of the circuit, and its VCC to +5V.
  f. Connect the Chip Select (CS) pin of the SPI microSD card module to pin number 10 of the Arduino Mega 2560.
  g. Connect the Serial Clock (SCK) pin of the SPI microSD card module to the SCK pin of the Arduino Mega 2560 (i.e., pin 3 on In-Circuit Serial Programming (ICSP) header).
  h. Connect the Master Out Slave In (MOSI) pin of the SPI microSD card module to the MOSI pin of the Arduino Mega 2560 (i.e., pin 4 on ICSP header).
  i. Connect the Master In Slave Out (MISO) pin of the SPI microSD card module to the MISO pin of the Arduino Mega 2560 (i.e., pin 1 on ICSP header). The final circuit should look like as in Figure 5.

***Note:*** The circuitry shown in Figure 5 represents the complete hardware architecture underlying the proposed sensing technology. It comprises a 3S1P 12 V battery as the power source, stacked PCB signal processing units, an RTC module, an SPI-based microSD module, and the Arduino Mega 2560. Despite the relatively high component count, the system was tested and verified to be fully installable within the enclosure of the sensor box illustrated in Figure 3.

***Note:*** Each of the DRV5055A1 is to be fixed inside the two adjacent fastening tubes of the sensor box shown in Figure 1. The 3-wire terminal of each DRV5055A1 (i.e., Vcc, GND, Vout) is connected to each SPU through header connectors. The cabling between the DRV5055A1 and each SPU is held internally inside the sensor box of Figure 3.

### Arduino programming

**Timing: 60 min**

In this second major step, the Arduino Mega 2560 is programmed to receive the MF-to-voltage values received from the SPUs, and to store them on the SD card with timestamps from the RTC.

***Note:*** Please refer to the troubleshooting 10 to acknowledge the probabilistic failure associated in this step with its potential solutions.

7. Start with library inclusion and variables definition.
  a. Include the libraries SPI.h, SdFat.h, RTClib.h.
  b. Define SD card pin, filename, sensor pin, sampling count, reference voltage and Analog to Digital Conversion (ADC) resolution.
  c. Create objects for SD card, file handling, and RTC.

***Note:*** The following code snippet represents the above-described steps with only 1 sensor connected to A0 (as example). The intrinsic design^1^ utilizes 8 MF sensors installed in the adjacent fastening tubes of Figure 3.

>#include <SPI.h>

>#include <SdFat.h>

>#include <RTClib.h>

>#define CS_PIN 10

>#define FILE_NAME "data.csv"

>SdFat SD;

>File file;

>RTC_DS3231 rtc;

>const int sensorpin = A0;

>const int samplecount = 1000;

>const float vref = 5;

>const float resolution = 1024;

>int analogvalue = 0;

>float voltage = 0;

***Note:*** The choice of ‘samplecount’ ensures statistical reliability and noise reduction by averaging transient fluctuations in the analog signal. It additionally increases the chance of capturing the true peak voltage while filtering short-term noise, leading to a more accurate and stable ‘voltage’ reading.

8. System initialization.
  a. Start serial communication.
  b. Initialize RTC. Break operation when RTC is not found.
  c. Initialize SD card. Break operation when SD card is not found.
  d. Create a CSV file in which the current norms are to be stored.
  e. Set sensor pin as input.

>void setup() {

>Serial.begin(9600);

>if (!rtc.begin()) {

>Serial.println("unable to find RTC");

>while (1);

>}

>if (!SD.begin(CS_PIN)) {

>Serial.println("SD card initialization failed");

>while (1);

>}

>Serial.println("SD card initialized.");

>if (!file.open(FILE_NAME, O_WRONLY | O_CREAT | O_APPEND)) {

>Serial.println("Opening file failed");

>while (1);

>} else {

>Serial.println("File opened successfully.");

>file.println("Time, Sensor Value");

>}

>pinMode(sensorpin, INPUT);

>}

9. Read sensor data.
  a. Read number of samples from the sensor.
  b. Convert ADC values to voltages.
  c. Detect and store the maximum voltage.

***Note:*** Since the DRV5055A1 is intended for application in the space near overhead TLs, in which flowing currents are of alternating nature, the produced output voltage would also inherit this alternating nature. Therefore, the maximum voltage represents the actual current norm, in comparison with the DC producible voltage by the same sensor in the nearfield of a permanent magnet.

>float readanalog(int pin) {

>float maxvoltage = 0.0;

>for (int i = 0; i < samplecount; i++) {

>analogvalue = analogread(pin);

>voltage = (analogvalue ∗ vref) / resolution;

>if (voltage > maxvoltage) {

>maxvoltage = voltage;

>}

>delayMicroseconds(100);

>}

>return maxvoltage;

>}

10. Data saving of the sensor data.
  a. Read sensor data.
  b. Get the actual time information from RTC and format is as “hh:mm:ss”.
  c. Write time and sensor value to the originally created CSV file in step 7 under this second major step.
  d. Add a delay before taking the next read.

>void loop() {

>float sensorvalue = readanalog(A0);

>DateTime now = rtc.now();

>String currenttime = String(now.hour()) + ":" + String(now.minute()) + ":" + String(now.second());

>file.print(currenttime);

>file.print(", ");

>file.println(sensorvalue);

>file.flush();

>Serial.print("Time: ");

>Serial.print(currenttime);

>Serial.print(" - Sensor value: ");

>Serial.println(sensorvalue);

>delay(1000);

>}

### MATLAB programming

**Timing: 60 min**

With the saved values on the SD card, representing the RMS current norms, measured in a defined time window, in this third major step, the sensed RMS norms are visualized versus time in MATLAB R2023a.

11. Define and check the CSV file.
  a. Set ‘data.csv’ as the filename.
  b. Verify if the file exists; proceed when true and open it; raise an error when false and abort.

>filename = 'data.csv';

>if exist(filename, 'file')

>fid = fopen(filename, 'rt');

>headerLine = fgetl(fid);

⋮

>else

>error('data.csv file does not exist in the current directory');

>end

12. Read the data from the CSV file then close it.

>data = textscan(fid, '%s%f', 'Delimiter', ',', 'HeaderLines', 0);

>fclose (fid);

13. Extract time and sensor values then check if both are of the same length.

>timestrings = data {1};

>normvalues = data {2};

>if length(timestrings) ∼= length(normvalues)

>error (‘Time and sensor values are of different length’);

>end;

14. Convert time strings to data time format.

>time = datetime(timestrings, 'InputFormat', 'HH:mm:ss');

15. Initialize the array of the converted values representing the RMS current norms.

>convertedvalues = zeros(size(normvalues));

16. Map the original normvalues to convertedvalues.
17. Plot the data with converted normvalues.

>figure;

>plot (time, convertedvalues, ‘-o’);

>xlabel (‘Time’);

>ylabel (‘Current norm [Amps]’);

>title(sprintf('Sensor Data - %s', datestr(now, 'dd-mmm-yyyy, dddd')));

>grid on;

## Expected outcomes

**Figure 6:**
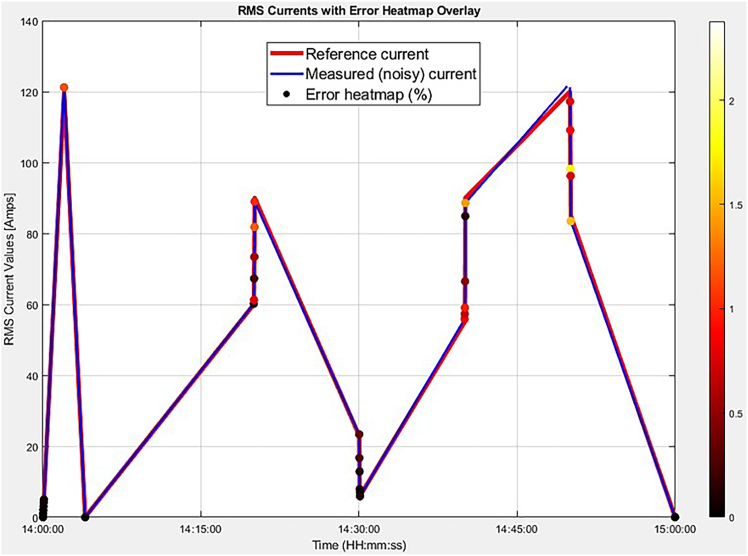
Heatmap error representation of reference (clean) vs. measured (noisy) currents

The application of this protocol, encompassing the physical circuitry of the DRV5055A1 integrated with the designed SPUs, alongside the software implementation in Arduino IDE and MATLAB, facilitates contactless, offline current monitoring and analysis. The 3S1P (12 V) 5000 mAh battery enables continuous operation of the sensor box, as depicted in Figure 1, for a duration of six hours. This enables the proposed sensor to capture the RMS current values over a 6-h period while suspended on the overhead TLs. The designed SPUs ensure stable and safe voltage levels for input to the Arduino Mega 2560 Analog Inputs (AIs). The RTC provides accurate timekeeping, supported by a backup battery with a theoretical lifespan of up to one year. The data stored on the SD card amounts to less than 2 kilobytes.^1^ In MATLAB, the step-by-step code implementation provided in the third major step allows for automatic data visualization, plotting the RMS current values against the actual time of measurement, with the simple insertion of the SD card via Universal Serial Bus (USB) to execute the script. The physical current sensor should exhibit a high accuracy, with a maximum absolute voltage deviation of 10 mV at 120 ARMS, with deviations below 0.5 mV for currents under 8 ARMS, indicating minimal error at low current levels. Over the entire measurement range of [0;120] ARMS, the overall error would quantify at 1.7%, corresponding to a current measurement accuracy of 98.3%, even in the presence of 20% total harmonic distortion and injected harmonics at 150 Hz and 250 Hz: with that being said, Figure 6 provides a visual representation of the sensor’s measurement accuracy over a continuous one-hour testing period, during which the current fluctuated randomly between 0 and 120 ARMS.

Furthermore, in 16 out of 32 measured points, the 4.88 mV threshold of the Arduino ADC would be expected to be exceeded (predominantly at currents ≥60 ARMS) implying that algorithmic compensation should be anticipated to enhance measurement integrity in mid- to high-range operating conditions. The observed and anticipated deviations are technically attributed to the nonlinear interaction between harmonic-induced flux density variations and the limited dynamic range of the DRV5055A1, particularly under localized field perturbations at elevated current levels. Additionally, the sensor’s temperature-dependent sensitivity (i.e., ranging from 100 mV/mT at 20°C to 115 mV/mT at 125°C) would be expected to introduce proportional bias errors due to thermo-magnetic expansion effects, with deviations potentially reaching 15%. These effects are further amplified by noise-induced micro fluctuations, likely penetrating the measurement signal as a consequence of suboptimal SPU filtering characteristics and limited electromagnetic shielding, especially at close sensor-to-conductor distances approaching saturation proximity. Overall, the protocol should offer an innovative current measurement technique with data storage on the SD card and visualization in MATLAB.

## Quantification and statistical analysis

The DRV5055A1 MF sensor is selected in a TO-92 package to ensure reliable and flexible installation within the fastening tubes depicted in Figure 1. One of the primary challenges in implementing the prototype proposed in this study is the constraint on overall design dimensions, particularly within the fastening tubes, which have a diameter of less than 2 cm. Each MF sensor is capable of detecting magnetic flux densities of up to ±21 mT. Consequently, at a 1 cm observation distance (considered as the measurement point for the magnetic field in the vicinity of the TL), each MF sensor reaches saturation at a current of 1050 A. Within each of the two adjacent fastening tubes, four MF sensors are integrated, denoted as Sensor 1 to Sensor 4. Sensor 2 serves as the reference sensor, meaning it is the starting point for ADC calculations, as it is positioned as close as possible to the TL behind the CF tube. When Sensor 2 reaches full saturation, the MATLAB algorithm triggers a flag, instructing the system to utilize the next closest MF sensor within the same tube. The result is then cumulatively added to the previously detected current of 1050 A (sensed by Sensor 2, which is now saturated). For integration with the subsequent SPUs, each DRV5055A1 sensor produces a maximum output voltage of less than 5 V at full saturation. Therefore, the input of each SPU is designed to accommodate voltage levels up to 5 V. The cutoff frequency of the LPFs is set to 55 Hz to suppress higher-order harmonics and noise signals, which typically accumulate in the TL’s operating environment and may otherwise compromise the integrity of the original signals captured by the MF sensors. The Arduino system at the final stage is adequately powered by the BD80GC0VEFJ-ME2 at +8 V. To ensure that the maximum threshold of the Arduino’s analog inputs is not exceeded, voltage regulation is achieved through the SPUs, specifically using voltage dividers. With a 10-bit ADC resolution, variations in the DRV5055A1 sensor output are precisely detected, allowing for accurate representation of the induced current magnitude. Data recording is managed using a 32 GB SD card, with data transmission facilitated via the SPI protocol through the ICSP header of the Arduino. The processed voltage values are timestamped using the DS3231MPMB1# RTC module and stored in a Microsoft Excel table, consisting of two columns: one for timestamps in the ‘hh:mm:ss’ format and the other for the corresponding filtered voltage values. This table is automatically saved onto the SD card and can subsequently be used to generate a visualization curve in MATLAB.

## Limitations

The study acknowledges several limitations. First, the cost efficiency of the proposed measurement solution remains an open consideration. Experimentation was limited to currents ≤120 ARMS due to the FLUKE 6105A Electrical Power Standard’s maximum output.^1^ As no alternative high-current sources were available, an extrapolation method was used, with the Arduino simulating higher currents and MATLAB generating graphical representations. The sensor box operates exclusively when drone-deployed onto TLs, reducing reliability and adaptability. Environmental impacts, particularly wind-induced disturbances affecting Hall-effect sensor positioning, were not tested. Consequently, accuracy under windy conditions remains unverified. Sag effects in overhead TLs may alter the calibrated positioning of Hall-effect sensors, leading to measurement inaccuracies. Additionally, exposure to strong electric fields induces voltages in the sensor box’s internal wiring, causing noise, distortion, and potential sensor malfunctions, particularly in high-voltage TLs (132 kV–765 kV). Despite grounding, optimized PCB traces, and an LPF, additional shielding is required. Adjacent TLs also introduce signal interference, as MF sensors may detect multiple fields, reducing accuracy. The study lacks a shielding mechanism to mitigate this issue.

## Troubleshooting

### Problem 1

Incorrect pin mapping between symbol and footprint.

### Potential solution

• Double-check the datasheet and ensure each pin is correctly assigned before saving.
• Use EAGLE’s ERC to verify correct pin connections.

### Problem 2

Footprint dimensions do not match the physical component.

### Potential solution

• Cross-check the footprint dimensions against the component’s datasheet before finalizing.
• Print the footprint on paper and compare it physically with the component.

### Problem 3

Misalignment of layers during lamination.

### Potential solution

• Use fiducial markers and automated optical alignment systems to ensure precise layer stacking.
• Verify registration accuracy with X-ray inspection before final lamination.

### Problem 4

Insufficient plating thickness in Plated Through-Hole (PTH).

### Potential solution

• Monitor and control electroplating parameters, such as current density and bath composition.
• Perform cross-section analysis on test coupons to confirm plating thickness before proceeding.

### Problem 5

Defective solder mask application.

### Potential solution

• Ensure the PCB surface is clean and free of contaminants before solder mask application.
• Verify correct exposure settings and alignment to prevent incomplete pad openings.
• Conduct a final AOI inspection to detect missing or misapplied solder mask areas before proceeding.

### Problem 6

Uneven solder paste application.

### Potential solution

• Ensure the stencil is properly aligned and has the correct thickness for consistent paste deposition.
• Regularly inspect and clean the stencil to prevent clogging and uneven paste application.

### Problem 7

Misaligned component placement.

### Potential solution

• Use fiducial markers and optical alignment calibration to improve placement accuracy.
• Verify vacuum nozzle pressure and inspect for worn-out or damaged nozzles that could affect pick-and-place precision.

### Problem 8

Inconsistent reflow soldering results.

### Potential solution

• Regularly check and calibrate the oven’s temperature profile to match the solder paste specifications.
• Ensure proper ventilation and heat distribution to prevent cold solder joints or excessive thermal stress.

### Problem 9

Inspection failures leading to defective PCBs.

### Potential solution

• Fine-tune AOI settings to improve defect detection without generating false positives.
• Use X-ray inspection specifically for Ball Grid Arrays (BGAs) and hidden solder joints to catch potential issues early.
• Implement periodic manual sampling to verify AOI and X-ray results for accuracy.
• Cross-check electrical testing failures with visual and X-ray inspections to pinpoint root causes.

### Problem 10

The Arduino may fail to log data to the microSD card consistently because the file is opened in setup() but never closed or reopened in loop(). If the Arduino resets or loses power, data may not be saved properly, and the file could become corrupted.

### Potential solution

• Regularly close and reopen the file inside loop() before writing new data to ensure it is saved correctly and prevent corruption.
• Implement a fail-safe mechanism to detect microSD card write failures and reinitialize the microSD card if an error occurs, ensuring continued data logging.

## Resource availability

### Lead contact

Further requests for resources and materials should be directed to and will be fulfilled by the lead contact, Detlef Schulz (detlef.schulz@hsu.hamburg).

### Technical contact

Technical questions on executing this protocol should be directed to and will be answered by the technical contact, Khaled Osmani (alosmani.k@hsu-hh.de).

### Materials availability

This study did not generate new unique reagents.

### Data and code availability

The published article includes all code generated or analyzed during this study.

## Acknowledgments

This research paper is part of the project DNeD (“Digitalisierte, rechtssichere und emissionsarme flugmobile Inspektion und Netzdatenerfassung mit automatisierten Drohnen,” engl. “Digitalised, legally safe and low-emission airborne inspection and grid data acquisition using automated drones”) and was funded by dtec.bw—Digitalization and Technology Research Center of the Bundeswehr. dtec.bw is funded by the European Union—NextGenerationEU.

## Author contributions

K.O. was responsible for preparing the initial manuscript draft, designing the electronic boards, developing and implementing the algorithms, and conducting laboratory testing of the complete sensor unit. D.S. supervised the project, oversaw its administration, and verified the results as well as the scientific validity of the work.

## Declaration of interests

The authors declare no competing interests.
